# Solvent fractions of selected Ethiopian medicinal plants used in traditional breast cancer treatment inhibit cancer stem cells in a breast cancer cell line

**DOI:** 10.1186/s12906-020-03154-5

**Published:** 2020-11-25

**Authors:** Nigatu Tuasha, Daniel Seifu, Endalamaw Gadisa, Beyene Petros, Stina Oredsson

**Affiliations:** 1 Department of Microbial, Cellular and Molecular Biology, College of Natural and Computational Sciences, Addis Ababa University, Addis Ababa, Ethiopia; 2 Armauer Hansen Research Institute (AHRI), Addis Ababa, Ethiopia; 3 Department of Biology, Faculty of Natural Sciences, Lund University, Lund, Sweden; 4 Department of Biochemistry, School of Medicine, College of Health Sciences, Addis Ababa University, Addis Ababa, Ethiopia

**Keywords:** Breast cancer, Cancer stem cells, Ethiopia, JIMT-1, Traditional medicine

## Abstract

**Background:**

The incidence and mortality of breast cancer in women is increasing worldwide. Breast cancer contains a subpopulation of cells known as cancer stem cells (CSCs). The CSCs are believed to be responsible for chemotherapeutic resistance and are also involved in tumor initiation, progression, evolution, and metastasis to distant sites. The present study aimed to investigate the anti-CSC potential of selected Ethiopian medicinal plants traditionally used for breast cancer treatment.

**Methods:**

The solvent fractions of three medicinal plants (the ethyl acetate fraction of *Vernonia leopoldi,* the aqueous fraction of *Sideroxylon oxyacanthum,* and the chloroform fraction of *Clematis simensis*) resulting from the methanolic crude extracts were selected based on their previously demonstrated cytotoxic effects on breast cancer cell lines. The effect of these solvent fractions on the status of the cancer stem cell subpopulation of the JIMT-1 cell line was assessed by flow cytometric evaluation of the proportion of aldehyde dehydrogenase positive cells and by measuring colony forming efficiency in a serum-free soft agar assay after treatment. Effects on cell migration using a wound healing assay and on tumor necrosis factor-α-induced translocation of nuclear factor-kappa B to the cell nucleus were also investigated.

**Results:**

The solvent fractions showed a dose-dependent reduction in the aldehyde dehydrogenase positive subpopulation of JIMT-1 cells. The chloroform fraction of *C. simensis* (80 μg/mL) completely blocked colony formation of JIMT-1 cells. The wound healing assay showed that all fractions significantly reduced cell migration. The ethyl acetate fraction of *V. leopoldi* (0.87 μg/mL) significantly inhibited tumor necrosis factor-α-induced nuclear factor-kappa B translocation to the nucleus.

**Conclusion:**

The solvent fractions of the medicinal plants showed desirable activities against breast cancer stem cells in the JIMT-1 cell line, which warrants further studies.

## Background

Breast cancer is the most commonly diagnosed malignancy in women worldwide, with an estimated 2.09 million new cases reported in 2018 [1]. It affects approximately 25% of women in both the developed and less developed world [2, 3]. It remains the number one cause of death among females in less developed countries and is the second leading cause of cancer death among females in more developed countries, next to lung cancer [3, 4].

Breast cancer tissue contains a small population of highly tumorigenic multi-potential cells with self-renewal properties known as cancer stem cells (CSCs) [5]. Although the presence of CSCs was first reported in myeloid leukemia, they are now found in most solid tumors [6–9]. CSCs are very rare but are thought to be the main drivers of tumor growth and metastases. Much evidence points to CSCs being refractory to available cancer treatment regimens and thus contributing to cancer relapse and metastases [10, 11]. There are several phenotypic markers for breast CSCs. These include the expression of cluster of differentiation (CD)44 and absence of CD24 expression on the cell surface and the presence of enhanced aldehyde dehydrogenase (ALDH) activity [5, 9]. Cell migration, one of the characteristic features of CSCs, is a prerequisite for cancer metastases, which results in tumor initiation at different sites of the body [12, 13]. One of the important signaling pathways maintaining CSCs is the nuclear factor-kappa B (NF-κB) pathway. Aberrant NF-κB activation has been shown to be involved in breast CSC phenotypic features by cross-talking to several other signaling pathways [14]. In most breast cancers, the NF-κB pathway is activated constitutively and plays a critical role in cell survival, proliferation, and inflammation [15]. NF-κB is found in the cytoplasm in an inactive form associated with the inhibitor of NF-κB (IκB) [16]. When it is released from the inhibitor, p65/NF-κB is translocated to the nucleus and activates gene transcription by binding to sequence-specific targets in DNA [17]. Therefore, it is an important target for screening potential anticancer agents.

In addition to finding compounds that kill cancer cells in general, searching for CSC targeting compounds is of importance. We have previously shown that solvent fractions of Ethiopian medicinal plants traditionally used in breast cancer treatment exert toxicity against a number of breast cancer cell lines at low concentrations evaluated using dose response assays [18]. Based on that study, we selected the most cytotoxic solvent fractions of the Ethiopian medicinal plants *Sideroxylon oxyacanthum* (Baill.) (Family: Sapotaceae), *Clematis simensis* Fresen. (Family: Ranunculaceae), and *Vernonia leopoldi* (Sch. Bip. ex Walp.) Vatke (Family: Asteraceae) for further investigation of their effects on CSCs. Here we present data on how treatment with these fractions affects the CSC subpopulation of the JIMT-1 breast cancer cell line.

## Methods

### The plants, extraction, and solvent-solvent fractionation

In a previous study, we determined the half maximal inhibitory concentration (IC_50_) of seven Ethiopian medicinal plants selected based on the recommendations of traditional medicine practitioners and on the frequency of use reports across the country [18]. Here, investigations were done on three solvent fractions with low IC_50_ values based on results obtained by treating various breast cancer-derived cell lines and one normal-like cell line [18]. Thus, here we investigate the effects of the ethyl acetate fraction of *V. leopoldi,* the aqueous fraction of *S. oxyacanthum*, and the chloroform fraction of *C. simensis* on CSC properties of the JIMT-1 breast cancer cell line. The IC_50_ of the ethyl acetate fraction of *V. leopoldi*, the aqueous fraction of *S. oxyacanthum*, and the chloroform fraction of *C. simensis* are 0.87 ± 0.2 μg/mL, 69 ± 2 μg/mL, and 80 ± 19 μg/mL, respectively, in the JIMT-1 cancer cell line [18]. The selectivity index of V. leopoldi, S. oxyacanthum and *C. simensis* are 1.98, 1.16, and 1.2, respectively, compared to the normal-like breast epithelial cell line MCF-10A [18].

The identification and solvent fractionation of the medicinal plants were done as described earlier [18]. Briefly, the medicinal plants were collected, and identification and authentication were done at the National Herbarium, Addis Ababa University, Addis Ababa, Ethiopia. The voucher specimens were deposited at the herbarium with voucher numbers NT014 (*S. oxyacanthum*), NT037 (*C. simensis*), and NT073 (*V. leopoldi*). Crude methanolic extraction (80% methanol) was carried out by maceration using a rotary water bath shaker (DZK-2, Shanghai, China) (120 routes per minute) for 72 h at 21 °C. The extract was concentrated using a rotary vacuum evaporator (BÜCHI, Germany) under reduced pressure at 45 °C, and then the sample was freeze-dried by lyophilization (CHRIST, Alpha 2–4 LDplus, Osterode, Germany). The solvent-solvent (1:1, v/v) partitioning was performed using a separation funnel. Briefly, the weighed amount of dried crude methanolic extract was dissolved in 250 mL of 10% methanol in Millipore H_2_O in an Erlenmeyer flask. An equal volume (i.e. 250 mL) of hexane (100%) was added, the solution gently mixed in the funnel, and the solution kept at room temperature for 1 h to allow the formation of aqueous and hexane layers. After successfully recovering the aqueous phase, an equal volume of chloroform (100%) was added, gently mixed, and kept at room temperature for 1 h to allow the formation of aqueous and chloroform layers. The same procedure was repeated with the solvent ethyl acetate (100%) and ethyl acetate and aqueous phases were separated. All the fractions were concentrated using a rotary vacuum evaporator and freeze-dried by lyophilization [18]. The solvent fractions were labeled and kept at − 20 °C until use. All chemicals used for the extraction and partitioning process were purchased from Sigma-Aldrich (St. Louise, MO, USA).

### Culturing condition for the cell line

The JIMT-1 cell line [19] (ACC589) was purchased from the German Collection of Microorganisms and Cell Cultures (Braunschweig, Germany). Upon receipt, the cells were thawed and amplified, and ampules were frozen in a liquid nitrogen container. The JIMT-1 cells were routinely cultured in DMEM/Ham’s F-12 medium (VWR, Lund, Sweden) supplemented with 10% fetal bovine serum (FBS) (VWR), 1 mM non-essential amino acids (VWR), 10 μg/mL insulin (Sigma-Aldrich, Stockholm, Sweden), 1 mM L-glutamine (VWR), and 100 U/mL penicillin/100 μg/mL streptomycin (VWR). The JIMT-1 cells were seeded at the density of 0.015 × 10^6^ cells/cm^2^ in tissue culture vessel containing 0.2–0.3 mL per cm^2^ of medium, and then kept at 37 °C in a humidified incubator with 5% CO_2_ in air. After 4 days of culturing, the cell density was around 0.133 × 10^6^ cells/cm^2^, i.e. the cells had gone through approximately 3 population doublings. The lag phase of the cells after trypsinization is 24 h, giving a population doubling time of around 24 h. The JIMT-1 cells were kept under strict surveillance for these proliferation characteristics and also morphology and only used for 50 passages and then a new ampoule was thawed. The cells were tested for mycoplasma and were found to be negative (Eurofins GATC Biotech, Konstanz, Germany).

### Breast CSC population estimation by an ALDH assay

The JIMT-1 cells were seeded in Petri dishes at the density described above and incubated for 24 h. The solvent fractions were added to their final concentrations with dose ranges between IC_25_ and IC_50_ values, and the cells were incubated for 72 h. Accutase™ (Stem Cell Technologies, Grenoble, France) was used to detach the cells, and the detached cells were collected in PBS containing 1% FBS and counted using a hemocytometer. The ALDEFLUOR™ kit (Stem Cell Technologies, Grenoble, France) procedures as given in the manufacturer’s protocol were followed. The BD Accuri C6 Flow cytometer (BD Biosciences, San Jose, CA, USA) was used to analyze the samples. DEAB-treated cells for each sample were used to set the ALDH^+^ region. The data was evaluated using CFlow software.

### Colony forming efficiency assay

The effect of the solvent fractions on colony forming efficiency (CFE) was evaluated using a serum-free soft agar assay. The JIMT-1 cells were seeded in Petri dishes at the density described above and incubated for 24 h. The solvent fractions of the extracts were added to the final concentrations of 0.633 μg/mL to 80 μg/mL (IC_25_ and IC_50_ included), while the controls were treated with 0.2% methanol in PBS (i.e. the same final methanol concentration as for the solvent fractions). The Petri dishes were incubated at 37 °C in a humidified incubator with 5% CO_2_ in air for 72 h. The cells were detached through treatment with Accutase™ for 10 min at 37 °C and kept on ice while determining the cell concentration by counting in a hemocytometer. MEBM basal medium containing hydrocortisone, insulin, epidermal growth factor (CC-4136 kit, Cambrex, Walkersville, Maryland, USA), B27 supplement (Thermo Fisher Scientific), basic fibroblast growth factor (R&D Systems, Minneapolis, MN, USA (20 ng/mL), penicillin (100 U/mL), and streptomycin (100 μg/mL) was heated to 42 °C and mixed with agarose to a final concentration of 0.4%. The cells were added to a final concentration of 1000 cells/mL. Then, 500 μL of this mixture was immediately added to the inner wells of hydrophobic 48-well plates, and the outer rows of the plates were filled with 1 mL PBS to minimize evaporation. The plates were wrapped in plastic wrap and incubated at 37 °C in a humidified incubator with 5% CO_2_ in air for 14 days. The colonies were counted visually in an inverted phase-contrast microscope using the 10x objective.

### Wound healing assay

The JIMT-1 cells were seeded at a high density (125,000 cells/cm^2^) in Petri dishes (3 cm diameter) and incubated at 37 °C in a humidified incubator with 5% CO_2_ in air for 24 h to allow them to attach and form a confluent layer of cells. Then, the medium was removed, and three parallel scratch wounds (left, middle, and right side of the dish) were made in the cell layer using a sterile 200 μL pipette tip [20]. The cell layer was washed twice with PBS and serum-free medium was added to minimize the influence of cell proliferation on the wound healing process. The respective solvent fractions were added to the final concentration of dose ranges, including IC_50_ and IC_25_ concentrations, while the controls were treated with 0.2% methanol in PBS. Images of the wound areas were taken immediately before the incubation at 37 °C in the CO_2_ incubator (time 0). The wound area was photographed at 24, 48, and 72 h after time 0. To estimate the wound closure, the scratch areas were measured with ImageJ 1.47v software. The wound closure was calculated as percentage of the wound at time 0.

### Inhibition of p65/NF-κB translocation

We evaluated the inhibition of the solvent fractions on TNF-α-induced translocation of cytoplasmic p65/NF-κB to the cell nucleus. For this experiment, JIMT-1 cells were seeded (0.1 × 10^6^ cells/well) in 12-well plates with a round glass cover slip in each of the wells. The cells were then allowed to attach to the cover slips by incubating at 37 °C in the CO_2_ incubator for 48 h. After incubation, the cover slips were transferred to the wells of another 12-well plate containing 800 μL medium supplemented with 0.1% FBS and then incubated in CO_2_ incubator for 20 min to stabilize the temperature. The solvent fractions of the medicinal plants were added to the final concentrations of 0.87 μg/mL for the ethyl acetate fraction of *V. leopoldi*, 52 μg/mL for the aqueous fraction of *S. oxyacanthum*, and 80 μg/mL for the chloroform fraction of *C. simensis* and the plates were returned to the incubator for 1 h. Following this, TNF-α (25 ng/mL) was added and incubation continued for another 40 min. Then, the medium was removed, and the cells were fixed in 3.7% formaldehyde in PBS for 15 min and stored at 4 °C in 2 mL PBS. The cells were incubated in blocking buffer (1% bovine serum albumin [BSA] and 1% Tween 20 in PBS) for 1 h at room temperature to prevent nonspecific binding and to permeabilize the cells. Then, rabbit anti-p65/NF-κB primary antibody (ab76311) (Abcam, Cambridge, MA, USA) (diluted 1:250 in blocking buffer) was added, and the samples were incubated for 1 h at room temperature while being shaken gently. The samples were washed twice with PBS and then Alexa 488 anti-rabbit-conjugated secondary antibody (Molecular Probes, Inc., Eugene, USA) (diluted 1:500 in blocking buffer) was added. The plate was covered with aluminum foil, and the samples incubated for 1 h at room temperature while being shaken gently. Finally, the cover slips were mounted on glass slides using Mowiol as mounting medium (Sigma-Aldrich). The samples were kept at 4 °C and protected from light. The slides were imaged using an Olympus/Nikon epifluorescence microscope (Olympus Optical Co. Ltd., Japan) equipped with a digital camera (Nikon Imaging Japan Inc., Japan). The images were used for counting of the number of cells with positive nuclei in relation to all cells.

### Statistical analysis

All results are shown as mean ± SD. The data were analyzed by one-way analysis of variance (ANOVA) followed by a post-hoc Dunnett’s test. We consider *p* < 0.05 as statistically significant with a 95% confidence interval.

## Results

### Solvent fractions of the medicinal plants significantly reduce the ALDH^+^ populations of the JIMT-1 cells

**Fig. 1:**
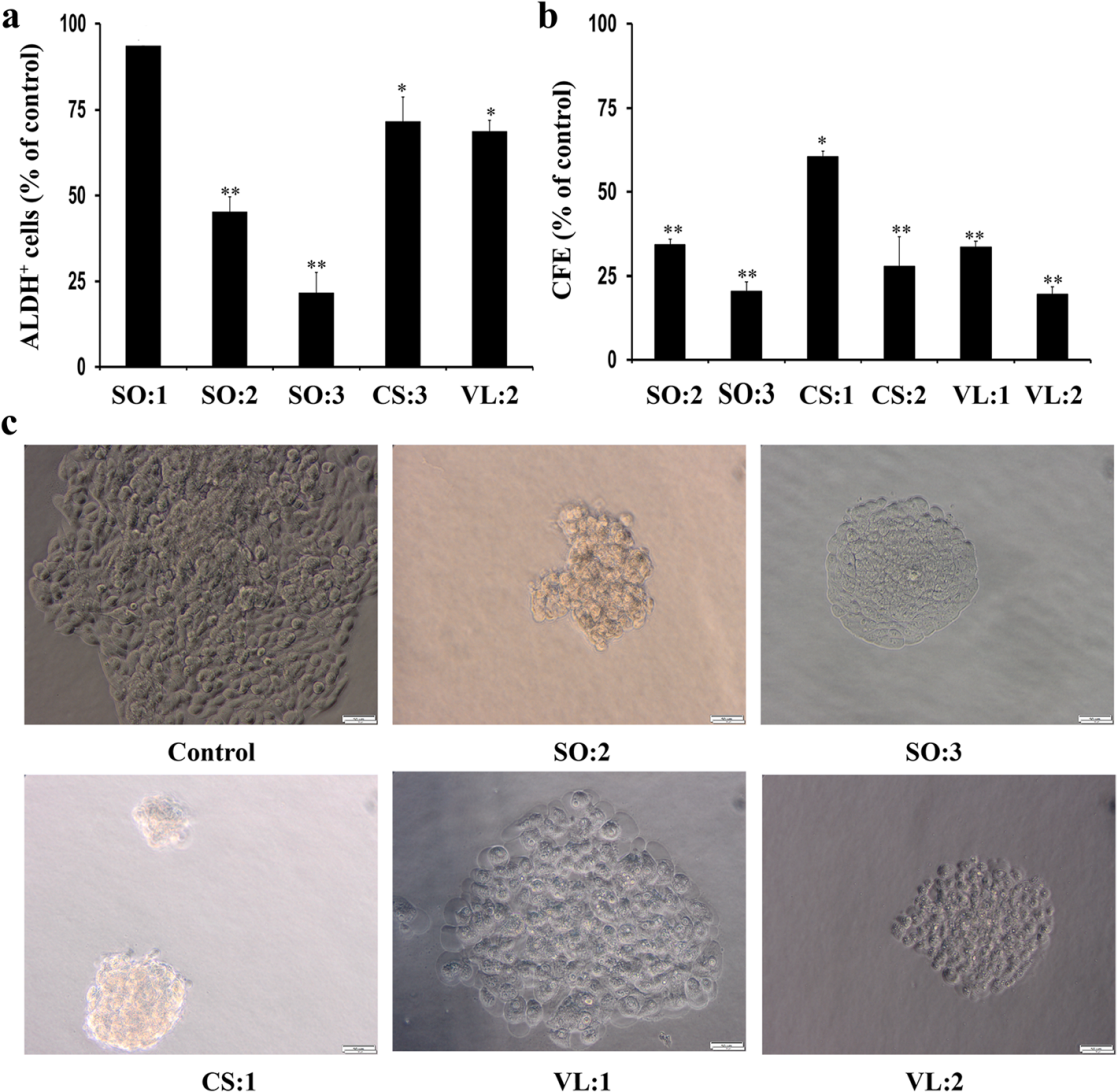
Treatment of JIMT-1 cells with different solvent fractions of Ethiopian plants reduces the ALDH^+^ CSC sub-population. **a** The ALDH^+^ population of JIMT-1 cells evaluated using flow cytometry after 72 h of treatment with the fractions. **b** Effects of the solvent fractions on CFE of JIMT-1 cells in a serum-free soft agar media incubated for 14 days. **c** Representative images showing the effects of different treatments on the sizes of the colonies in a serum-free CFE assay. *Keys:* aqueous fraction of *S. oxyacanthum* (SO: 1–20 μg/mL, SO: 2**–**52 μg/mL, SO: 3**–**69 μg/mL); chloroform fraction of *C. simensis* (CS: 1–15 μg/mL, CS: 2**–**34 μg/mL, CS: 3**–**80 μg/mL); ethyl acetate fraction of *V. leopoldi* (VL: 1–0.63 μg/mL, VL: 2–0.87 μg/mL). The data are presented as mean ± SD for *N* = 3 for each. *p < 0.05; **p < 0.001; CI: 95%; all the values were compared to control. Image bars = 50 μm

**Fig. 2:**
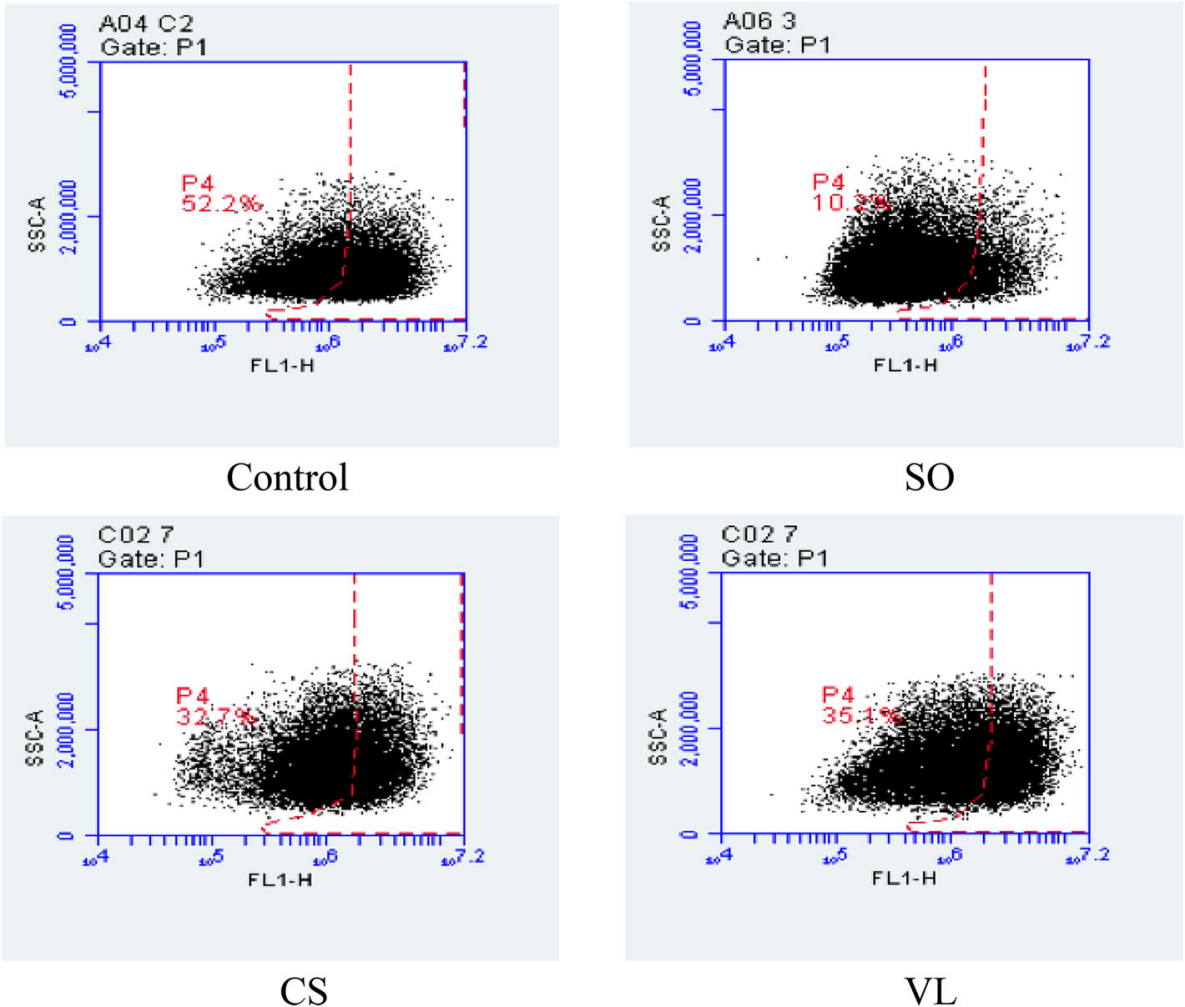
Representative cytograms of the flow cytometric analysis of ALDH^+^ populations. The ALDH^+^ population of JIMT-1 cells evaluated after 72 h of treatment with the fractions. SO, aqueous fraction of *S. oxyacanthum* (69 μg/mL). CS, chloroform fraction of *C. simensis* (80 μg/mL). VL, ethyl acetate fraction of *V. leopoldi* (0.87 μg/mL). FL1-H, green fluorescence signal - height. SSC-A, side scatter signal – area. The percentage shows the percent of total cells in the area delineated with red which defines the ALDH positive cells

One of the phenotypic markers for breast CSCs is enhanced ALDH activity [9], and thus the fraction of ALDH^+^ cells was estimated using the ALDEFLUOR™ assay. Treatment with the aqueous fraction of *S. oxyacanthum* at the IC_50_ concentration (SO:3, 69 μg/mL) and at a lower concentration (SO:2, 52 μg/mL) for 72 h significantly reduced the CSC population of JIMT-1 cells (*p* < 0.001) (Fig. 1a). However, treatment with the IC_25_ concentration (SO:1, 20 μg/mL) of the aqueous fraction of *S. oxyacanthum* did not result in reduction of the CSC population. Treatment with the chloroform fraction of *C. simensis* or with the ethyl acetate fraction of *V. leopoldi* at IC_50_ concentrations (CS:3, 80 μg/mL and VL:2, 0.87 μg/mL, respectively) also resulted in a significant reduction of the CSC population (*p* < 0.05) (Fig. 1a). Representative cytograms of the flow cytometric analysis of ALDH^+^ population are shown in Fig. 2.

### Colony forming efficiency

CFE in a serum-free medium is a functional assay used to investigate the survival of cells with stem-like properties [21]. Reducing the number of colony forming units is an important feature of potential chemotherapeutic compounds [22]. Treatment with the chloroform fraction of *C. simensis* at the IC_50_ concentration (80 μg/mL) resulted in 100% prevention of colony formation of JIMT-1 cells. Treatment with solvent fractions of *S. oxyacanthum* (SO:2 and SO:3), *C. simensis* (CS:1 and CS:2) and *V. leopoldi* VL:1 and VL:2) significantly reduced CFE in a dose-dependent manner compared to the controls (Fig. 1b). In addition to reducing the number of colonies, treatment with the fractions in general resulted in smaller colonies than in control (Fig. 1c).

### Treatment with the solvent fractions inhibits cell migration

**Fig. 3:**
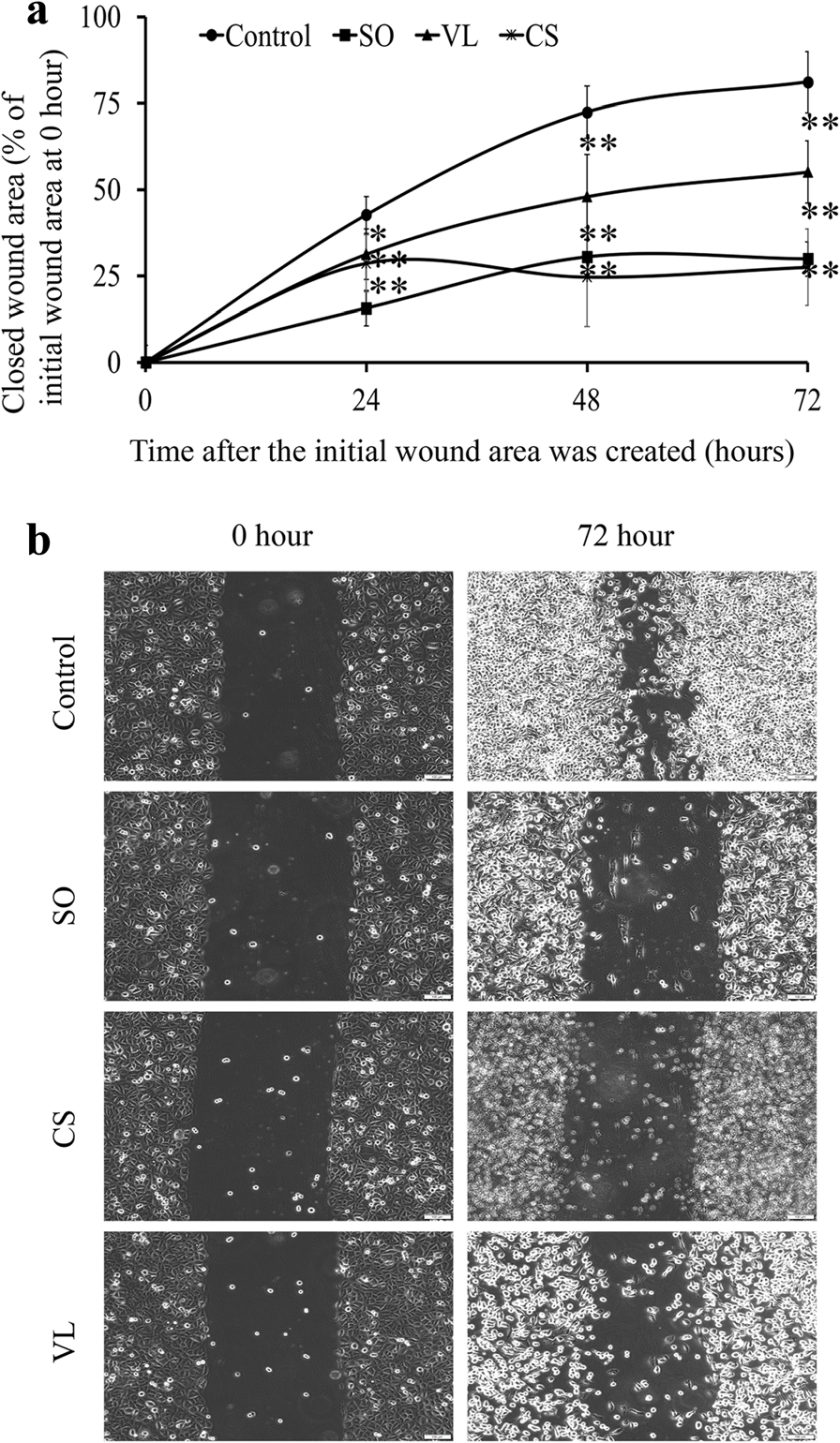
Treatment of JIMT-1 cells with different solvent fractions of Ethiopian medicinal plants reduces the migration. **a** Wound closure at 0, 24, 48, and 72 h. **b** Representative images taken by an inverted phase contrast microscope at 0 h and 72 h. SO, aqueous fraction of *S. oxyacanthum* (52 μg/mL). CS, chloroform fraction of *C. simensis* (80 μg/mL). VL, ethyl acetate fraction of *V. leopoldi* (0.44 μg/mL). The data are presented as mean ± SD with *n* = 3. *p < 0.05; **p < 0.001. Image bars = 100 μm

The aqueous fraction of *S. oxyacanthum* (52 μg/mL)*,* the chloroform fraction of *C. simensis* (80 μg/mL, i.e., IC_50_), and the ethyl acetate fraction of *V. leopoldi* (0.44 μg/mL) were evaluated in a wound healing assay. Treatment with the IC_50_ values of the ethyl acetate fraction of *V. leopoldi* and the aqueous fraction of *S. oxyacanthum* (i.e., 0.87 μg/mL and 69 μg/mL, respectively)*,* resulted in a large amount of cell death and interfered with wound area measurement. Based on the results of the wound closure, defined as 0% closure at 0 h for each sample, JIMT-1 cells treated with the chloroform fraction of *C. simensis* were arrested and unable to migrate after incubation for 24 h, when about only 27.5% wound closure was recorded, which was significantly less than control (*p* < 0.05) (Fig. 3). Treatment with the aqueous fraction of *S. oxyacanthum* resulted in a closure of 30.8% of the wound area at 48 h, and the inhibition was statistically significant compared to control at 48 h (*p* < 0.001). Treatment with the ethyl acetate fraction of *V. leopoldi* for 72 h resulted in 55.1% closure of the wound area. In the control, the wound closure was around 81% after 72 h of incubation, and the inhibition attained by treatment with the ethyl acetate fraction of *V. leopoldi* was significantly lower than in control (*p* < 0.001) (Fig. 3).

### Inhibition of NF-κB translocation

**Fig. 4:**
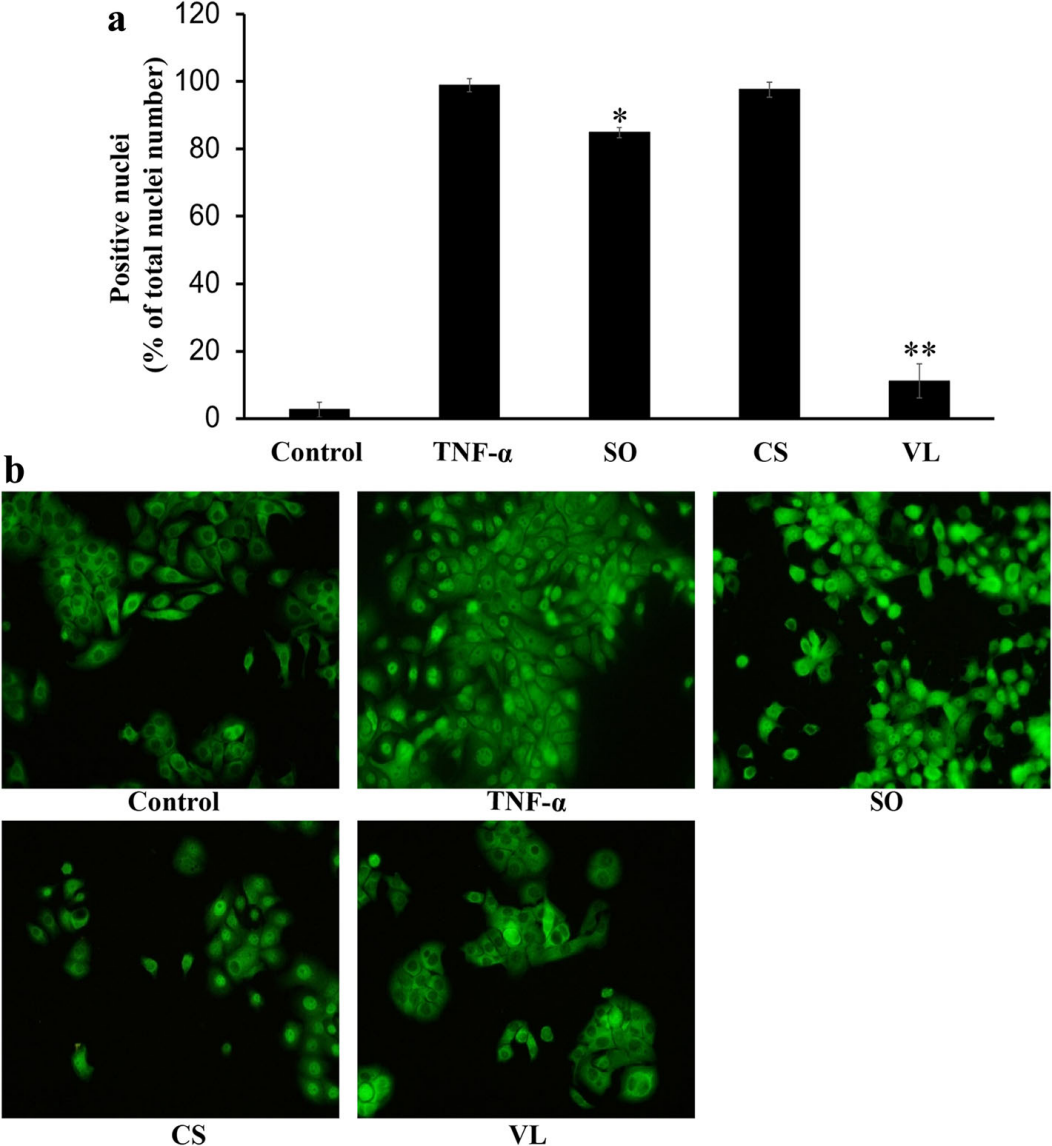
Treatment of JIMT-1 cells with different solvent fractions of Ethiopian plants inhibits TNF-⍺-induced translocation of p65/NF-κB to the nucleus. **a** p65/NF-κB positive nuclei in % of total nuclei number. The data are presented as mean ± SD with *n* = 3. *p < 0.05; **p < 0.001. **b** Representative images from independent experiments for each treatment. Control, no treatment. TNF-α, only treated with TNF-α. SO, aqueous fraction of *S. oxyacanthum* (52 μg/mL) and TNF-α. CS, chloroform fraction of *C. simensis* (80 μg/mL) and TNF-α. VL, ethyl acetate fraction of *V. leopoldi* (0.87 μg/mL) and TNF-α. To visualize the p65/NF-κB expression (green), the cells were fixed in formaldehyde and labeled as described in the Methods section. All images were taken with a 40x objective

In our experiment, a pro-inflammatory cytokine, TNF-α, was used to activate the NF-κB pathway and induce translocation of p65/NF-κB from the cytoplasm to the nucleus [23]. If NF-κB is blocked by an inhibitor, there will be no nuclear translocation of p65/NF-κB after TNF-α treatment. In control, less than 3% of the nuclei were positive for p65/NF-κB and the number of positive nuclei increased to 99% after TNF-α treatment (Fig. 4). When the cells were treated with the ethyl acetate fraction of *V. leopoldi* (0.87 μg/mL), only 11% of the nuclei were NF-κB positive, a significant reduction compared to TNF-α treatment (*p* < 0.001). Treatment with the aqueous fraction of *S. oxyacanthum* (52 μg/mL) also reduced the number of NF-κB positive nuclei but to a lower extent than the ethyl acetate fraction of *V. leopoldi* (*p* < 0.05). Treatment with the chloroform fraction of *C. simensis* at 80 μg/mL did not reduce the TNF-α-induced p65/NF-κB translocation (Fig. 4).

## Discussion

Breast cancer is a complicated disease showing a high degree of heterogeneity associated with different clinical outcomes and treatment regimens [24]. The treatments range from only surgery, when the tumor is local, to combinations of surgery, radiation, chemotherapy, and targeted therapy depending on tumor size, metastasis, and molecular signatures [24, 25]. Except for surgery, almost any breast cancer treatment can result in resistance and a growing body of evidence points to CSCs as the source of resistance [5, 26, 27]. Breast CSCs are tumorigenic multi-potential cells with deregulated self-renewal properties [6]. Characteristically, these cells share many features in common with normal stem cells, such as a slow-division rate, self-renewal, and differentiation, together with other fundamental properties that increase their survival potential [10, 28]. Therefore, great effort is spent identifying the CSCs in a tumor and screening compounds that specifically target these cells. Natural products are a source of new compounds that should be investigated for their CSC-inhibiting activities.

One of the phenotypic markers for breast CSCs is expression of ALDH [9]. For instance, ALDH^+^ breast cancer cells were reported to be more tumorigenic, metastatic, invasive, and migratory compared to ALDH^−^ breast cancer cells and were associated with poor clinical outcomes and decreased patient survival [29, 30]. The significant reduction of ALDH^+^ CSC subpopulation following treatment with the ethyl acetate fraction of *V. leopoldi,* the chloroform fraction of *C. simensis*, or the aqueous fraction of *S. oxyacanthum* is in agreement with the earlier report based on flow cytometry [31], which showed the retention of fluorescent BODIPY-aminoacetate produced upon oxidation within the cells expressing high ALDH activity. This may imply that these plants used in TM can reduce the metastatic burden of cancer by specifically targeting the CSC subpopulation [32].

Another feature of CSCs is their ability to form colonies in serum-free medium [33]. The inhibition of colony formation in JIMT-1 cells that resulted in over 60% reduction of CFE upon treatment with 15 μg/mL of chloroform fraction of *C. simensis* shows the inhibition of the unlimited self-renewal property and the capacity to initiate and maintain malignancy of the CSCs [34, 35]. The dose dependent reduction in the number of colonies and reduced sizes of the colonies in comparison to the controls may indicate that the aqueous fraction of *S. oxyacanthum,* the chloroform fraction of *C. simensis*, or the ethyl acetate fraction of *V. leopoldi* have inhibitory effect against the features involved in maintaining the stemness of the CSCs. The finding is corroborated by a recent report that treatment of MCF7 and SKBR3 breast cancer cells with solvent extracts of *Viola odorata* reduced the colony formation and colony size in a soft agar assay [36].

Besides inhibiting CSCs specifically, as seen by the ALDH and CFE assays, treatment with the fractions at low concentrations also inhibited cell migration evaluated in a wound healing assay. In agreement with the present finding, various studies reported plant extracts with similar effects in various cancer cell lines, including MCF-7 and MDA-MB-231 cell lines [37, 38]. This finding is consistent with the report that an extract from *Galenia africana* inhibited the cell migration by downregulating the mesenchymal markers, vimentin and β-catenin, and upregulating E-cadherin, a well characterized epithelial marker [39]. Since cell migration involves the transition of epithelial cells into motile mesenchymal cells [40] and thus promotes CSC migration, contributing to the reconstitution of metastatic cancer at distant sites [41, 42], the present finding implicates that the bioactive fractions may contribute to reducing the metastatic burden of breast cancer.

The transcriptional activation of genes associated with cell proliferation, angiogenesis, metastasis, and suppression of apoptosis appears to lie at the heart of the ability of NF-κB to promote cancer therapy resistance [43, 44]. We here report that the ethyl acetate fraction of *V. leopoldi* significantly reduced the number of p65/NF-κB positive nuclei in TNF-α-treated cells compared to TNF-α treatment alone. As the NF-κB pathway is supposed to be important for CSC function, our data imply that the inhibitory effect of the solvent fractions on CSCs may partly be exerted through an effect on this pathway. This can be an indication that the molecular mechanism involved in CSC maintenance may have been affected. Interference with the NF-κB pathway has been demonstrated with a variety of secondary metabolites from plants [45]. The major classes of plant natural products that have been shown to interfere with the NF-κB pathway are phenolics, quinones, isoprenoids and derivative, and alkaloids.

## Conclusion

Here we have found that solvent fractions of traditionally used medicinal plants have anti-CSC activity in the JIMT-1 cancer cell line at a desirably low concentration. With these data, we urge other researchers to investigate if there is a similar anti-CSC activity in other cancer cell lines. This may aid in the recommendation of medicinal plants in the traditional treatment of cancer in areas of the world. The fact that the solvent fractions show activity at very low concentrations implies that plant extracts used in traditional medicine may have anti-CSC activity. It is of importance to expand these studies using several cancer cell lines to investigate if this is a general phenomenon affecting CSCs of different cancer types. Also, we suggest isolation of the specific active compounds from these fractions.

## Data Availability

The datasets used and/or analyzed during the current study are available from the corresponding author on reasonable request.

## Abbreviations

ALDH: Aldehyde dehydrogenase
BSA: Bovine serum albumin
CFE: Colony forming efficiency
CSCs: Cancer stem cells
FBS: Fetal bovine serum
NF-κB: Nuclear factor-kappa B
PBS: Phosphate-buffered saline
TNF-α: Tumor necrosis factor-α
